# Applications of Machine Learning for Cognitive Health in Older Individuals With HIV: Rapid Systematic Review

**DOI:** 10.2196/80433

**Published:** 2025-12-31

**Authors:** Hwayoung Cho, Jiyoun Song, Hannah Cho, Lin Li, Renjie Liang, Railton Miranda, Qianqian Song, Jiang Bian

**Affiliations:** 1 College of Nursing, University of Florida, 1225 Center Dr., Gainesville, FL, 32610, United States, 1 3522736598; 2 School of Nursing, University of Pennsylvania, Philadelphia, PA, United States; 3 College of Medicine, University of Florida, Gainesville, FL, United States; 4 School of Medicine, Indiana University, Indianapolis, IN, United States; 5 Regenstrief Institute, Indianapolis, IN, United States

**Keywords:** aging, HIV, dementia, cognitive impairment, machine learning, artificial intelligence, PRISMA, Preferred Reporting Items for Systematic Reviews and Meta-Analyses

## Abstract

**Background:**

More than half of people with HIV are now older than 50 years, and they face an approximately 60% higher risk of developing dementia compared with the general population. In recent years, the application of artificial intelligence, particularly machine learning, combined with the growing availability of large datasets, has opened new avenues for developing prediction models that improve dementia detection, monitoring, and management.

**Objective:**

This systematic review aimed to synthesize the existing literature on the application of machine learning in dementia research among older people with HIV and identify directions for future research.

**Methods:**

A comprehensive search was conducted in PubMed, CINAHL, and Embase in September 2024, limited to studies published within the past 10 years. Eligible articles included original research involving people with HIV applying at least 1 machine learning technique and reporting dementia-related outcomes.

**Results:**

The search yielded 721 articles, of which 26 (3.6%) met the inclusion criteria. Most studies were retrospective and conducted in the United States (n=14, 53.8%), primarily focusing on neurocognitive impairment and HIV-associated neurocognitive disorders. Supervised machine learning techniques were most frequently used and demonstrated strong predictive performance. Common methodological challenges included small sample sizes, lack of external validation, limited participant diversity, and concerns about biological interpretability and generalizability.

**Conclusions:**

Machine learning research on dementia among older people with HIV primarily targets HIV-associated neurocognitive disorders, with limited exploration of age-related neurodegenerative diseases such as Alzheimer disease and related dementias. The absence of longitudinal studies and external validation remains a key limitation. Future research should broaden the focus to all-cause dementia beyond HIV-specific conditions; apply advanced machine learning methods; and leverage large-scale longitudinal, multimodal datasets. Strengthening methodological rigor and enhancing real-world applications will be critical to improving early detection and effective management of cognitive health in this unique aging population.

## Introduction

Globally, there were approximately 39.9 million individuals living with HIV at the end of 2023 [1]. With advances in antiretroviral therapy, people with HIV are living longer, and more than 53% of them in the United States are now older than 50 years [2]. Although “older adults” commonly refers to individuals aged 65 years and above, research in the context of HIV often considers aging-related health concerns to emerge earlier due to accelerated and accentuated aging processes. There is a high frequency of neurocognitive decline reported in people with HIV, with approximately a 60% higher risk of developing dementia as they age compared with the general population [2]. Given that there is no cure for dementia, early detection and effective management are critical [3].

However, developing early diagnostic and effective management tools for dementia in this population presents unique challenges. People with HIV exhibit distinct neurological changes, which include not only Alzheimer disease (AD) and AD-related dementias (ADRD; such as vascular dementia, Lewy body dementia, frontotemporal dementia, and mixed dementia) but also HIV-associated neurocognitive disorders and HIV-associated dementia. The complexity of HIV-related pathologies and comorbidities, along with inconsistent reporting, has posed significant challenges in HIV and aging research [4-6].

Despite this elevated risk, existing dementia detection and management strategies often fail to address the unique needs of people with HIV. In recent years, the application of artificial intelligence, particularly machine learning, combined with the increasing availability of large datasets, has opened new avenues for developing prediction models to improve dementia detection, progress monitoring, and management [3,7-11], offering promising alternatives for unraveling these complex relationships. Although the number of studies leveraging machine learning in predictive modeling of dementia outcomes in people with HIV has been growing, the scientific literature still lacks a synthesized review focused on this population. To address this gap, we conducted a rapid systematic review that followed the core principles of systematic review methodology but omitted a formal study quality appraisal to expedite evidence synthesis [12-14]. The objective of this review was to synthesize the existing literature that has applied machine learning in dementia research for people with HIV and to highlight directions for future research.

## Methods

This systematic literature review followed the Preferred Reporting Items for Systematic Reviews and Meta-Analyses (PRISMA) statement [15].

### Data Sources and Search Strategy

A comprehensive search strategy was developed by the authors (Hwayoung Cho, LL, and RL) through a literature review, with assistance from a health sciences librarian at the academic institution where the study was conducted. Using 3 databases (ie, PubMed, CINAHL, and Embase), we searched the literature on September 24, 2024, with a 10-year publication limit to ensure inclusion of the most current advancements in artificial intelligence and machine learning applications in this area of research. To capture all relevant studies, additional hand searches were also conducted using reference lists obtained from the literature relevant to this review. The search terms used in the title, abstract, or keywords included the following themes: HIV infections; all-cause dementia including AD and ADRD, HIV-associated dementia, and HIV-associated neurocognitive disorders; machine learning approaches including artificial intelligence, machine learning, and deep learning. Medical Subject Headings (MeSH) terms for PubMed, CINAHL Headings for CINAHL, Emtree terms for Embase, and free-text terms were used with the Boolean searching technique. Details on the search strategies are presented in Multimedia Appendix 1.

### Overview of Machine Learning Techniques

To provide context, we briefly summarize the most common machine learning techniques [11,16,17]. Supervised learning approaches, such as support vector machines (SVMs) [18,19], random forests, and logistic regression, use labeled data to classify or predict outcomes. Unsupervised methods, such as clustering, identify hidden patterns in unlabeled data. Semisupervised methods combine these two approaches, while deep learning techniques [20], including convolutional neural networks [21] and deep neural networks [22], use multilayered neural architectures to capture complex nonlinear patterns. These approaches are increasingly applied in health care research to improve diagnostic accuracy and risk prediction [11,16,17].

### Study Selection and Eligibility Criteria

A web-based collaboration software platform that streamlines the production of systematic reviews, Covidence (Veritas Health Innovation), was used to facilitate the title and abstract screening, full-text review, data extraction, conflict resolution, and data verification. During each screening and review stage, titles and abstracts (LL, RL, RM, JS, and Hannah Cho) and full texts (JS and Hannah Cho) were independently assessed by two reviewers to determine study eligibility. To assess interrater reliability during the screening and review phases, the Cohen κ was calculated. Differences were resolved by consensus and, if necessary, by a sixth reviewer (Hwayoung Cho).

The inclusion criteria included an original research article that (1) focused on people with HIV as the target study population, (2) examined at least one dementia-related outcome, and (3) applied at least one machine learning technique and reported evaluation metrics (eg, area under the curve [AUC] of the receiver operating characteristic, sensitivity, specificity, precision, and *F*_₁_-score). We excluded studies that examined only mild cognitive impairment without including any form of dementia, as our focus was on research specifically involving dementia-related conditions. We also excluded studies that were not available in the English language or full text, or gray literature (eg, clinical trials registries, conference abstracts, dissertations or theses, government reports, issues papers, letters, comments, editorials, correspondences, blogs, or newsletters).

### Data Extraction and Synthesis

The following information was extracted from the studies included in the final review using a Microsoft Excel spreadsheet: last name of the first author, year of publication, location of the study conducted, study objective, study design, study sample (eg, demographics and sample size), data source, data period, and subtype of dementia focused. We also synthesized the following information: main aim of using machine learning, type of machine learning techniques, evaluation metrics used for machine learning models, key outcomes from machine learning models, and machine learning–related limitations reported in the article. Adopting machine learning breakdown frameworks [11,17], we subcategorized machine learning techniques by how they infer patterns from data into the following five types: (1) supervised, (2) unsupervised, (3) semisupervised (ie, a combination of supervised and unsupervised), (4) reinforcement learning, and (5) deep learning. Consistent with rapid review methodology, we did not perform a formal study quality appraisal. Instead, we summarized the methodological challenges reported in the included studies. This approach aligns with the goal of rapid reviews to provide timely, systematic evidence synthesis using streamlined methods [12-14].

## Results

### Search Results

**Figure 1.**
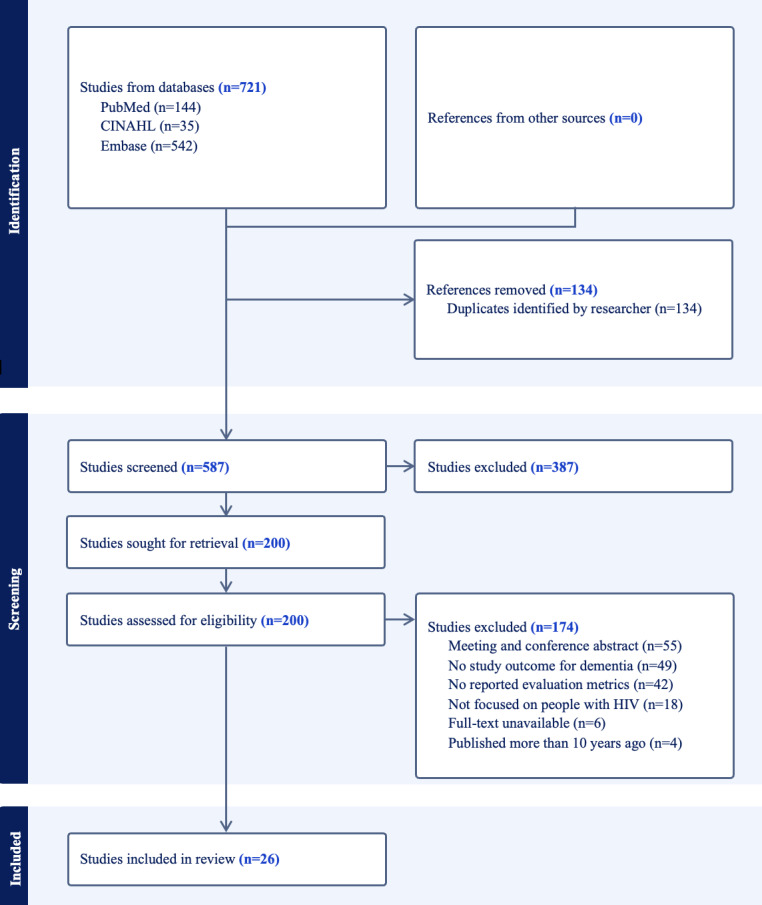
Preferred Reporting Items for Systematic Reviews and Meta-Analyses (PRISMA) flow diagram of the study selection process.

The initial searches resulted in 721 articles, including 144 (20%) from PubMed, 35 (4.9%) from CINAHL, and 542 (75.1%) from Embase. After removing duplicates, 587 (81.4%) articles were screened for titles and abstracts, and 200 (34.1%) articles were reviewed with full texts based on our eligibility criteria. We had high agreement between raters when making review decisions (Cohen κ≥0.80). A total of 26 (13%) studies that met the criteria were included in the review. The PRISMA flow diagram illustrating the review process is depicted in Figure 1.

### Study Characteristics

An overview of study characteristics for a total of 26 studies is presented in Multimedia Appendix 2. Most studies were conducted in the United States (n=14, 53.9%) [23-36], followed by China (n=5, 19.2%) [37-41]; Canada (n=3, 11.5%) [42-44]; and other countries (n=4, 15.4%) including Colombia [45], Brazil [46], Ethiopia [47], and Japan [48].

The majority of studies used retrospective observational designs, including 3 (11.5%) cohort studies [25,27,42] and 2 (7.7%) cross-sectional [45,47] studies, while 21 (80.8%) did not specify their design. Data sources included neuroimaging (magnetic resonance imaging [MRI] and functional MRI [fMRI]) [26,28,29,31,32,34,39-41,44], neuropsychological tests, clinical and demographic variables from electronic health records and research cohorts [27,38,40,43,44,47], and other specialized data such as substance use questionnaires [25], the Center for Epidemiological Studies Depression Scale [25,46], and biological samples from research programs [25,30,31]. Cognitive measures varied across studies and included the Mini-Mental State Examination [26,45], the International HIV Dementia Scale [47], self-reported memory assessments [25,31], and comprehensive neuropsychological test batteries [36,41,43].

The studies examined multiple forms of cognitive impairment among people with HIV, including neurocognitive impairment [26-28,38,42,46,47] and HIV-associated neurocognitive disorders [23-25,29,30,34,39,44,45,48], with particular attention to asymptomatic neurocognitive impairment as a subset of HIV-associated neurocognitive disorder [37,40,41]. Control groups included healthy individuals [26,28,35,38-40,48], HIV-negative cognitively normal participants [24,31,37,39], preclinical HIV-asymptomatic neurocognitive impairment cases [41], and neuropsychologically normal controls [23,30,33,43].

Sample sizes ranged from 9 to 8490 participants, with ages spanning 21 to 81 years. Almost half of the 26 studies (n=12, 46.2%) focused on middle-aged adults (40‐50 years) [24-27,32,33,36,42-46]. Most participants were male (>70%) [23,24,26,28,31-33,36,37,42,44,46]. Several studies (n=4, 15.4%) reported that participants were predominantly White, followed by African American or Black individuals [30-32,42]. Two (7.7%) US studies included primarily African American samples (82.6 % and 69.1%‐89.8%) [27,33]. Hispanic participants were represented in some studies (n=4, 15.4%) [23,30-32], typically comprising 4.8% to 24% of samples [23,31]. A few studies (n=3, 11.5%) reported Asian or other racial groups, but these consistently accounted for less than 5% of participants [23,31,32].

### Key Findings on Machine Learning

An overview of main findings from the 26 studies is presented in Multimedia Appendix 3, summarizing machine learning applications in dementia research for people with HIV, the techniques and model performance used, and the limitations of these applications reported across studies.

### Applications for Machine Learning in Dementia Research Among People With HIV

In this review, machine learning was applied to improve the diagnosis, prevention, and management of dementia among older people with HIV across studies. Of the 26 studies, some (n=12, 46.2%) applied machine learning algorithms to enhance diagnostic accuracy by analyzing neuroimaging data, cognitive assessments, and biomarkers (including 1, 3.8% study focusing on genetic features [48]) to identify early signs of dementia in people with HIV [23,28-30,33,35,36,41,43,46-48], while other studies (n=2, 7.7%) used machine learning to monitor disease progression, identify symptom clusters, and optimize tailored treatment plans for people with HIV [38,45].

### Machine Learning Techniques and Model Performance

Various machine learning techniques were identified across the 26 included studies and classified into supervised, unsupervised, semisupervised (a combination of supervised and unsupervised), reinforcement learning, and deep learning [11,17]. Supervised machine learning was the most prevalent approach, applied in 20 (77%) studies [23,24,26,28-33,35-37,39-43,45-47]. Only 1 (3.8%) study applied an unsupervised machine learning approach [38], while a combination of supervised and unsupervised methods was used in 5 (19.2%) studies [25,27,34,44,48]. Deep learning was used in 2 (7.7%) studies [35,36], whereas reinforcement learning was not reported in any of the included studies.

Supervised machine learning techniques included SVMs, random forests, logistic regression, ensemble models (eg, Adaptive Boosting and Extreme Gradient Boosting), and feature selection methods such as least absolute shrinkage and selection operator and classification and regression trees [23,24,26,28-33,35-37,39-43,45-47]. SVMs were the most frequently applied algorithm, used in 7 (26.9%) studies [24,30,32,33,39,41,48], with reported AUC values up to 0.85 and classification accuracy as high as 82% [24]. Logistic regression was also implemented in 7 (26.9%) studies [25,26,28,37,43,46,47], achieving classification accuracy between 68% and 90% [43]. Random forests were used in 5 (19.2%) studies [31,42,43,46,48], demonstrating strong predictive performance with AUC values up to 0.87 and accuracy levels exceeding 80% [46]. Ensemble models, such as Adaptive Boosting, showed robust performance, with precision and recall scores of 0.80 and 0.77, respectively [46].

Unsupervised learning techniques, including k-means clustering, hierarchical clustering, and mutual connectivity analysis, were primarily used to stratify patients into subgroups and identify distinct cognitive profiles. For example, k-means clustering was applied to classify HIV-associated neurocognitive disorder subtypes and demonstrated strong performance in identifying connectivity profiles, achieving AUC values up to 0.89 [34].

Deep learning techniques were applied in only a few (n=2, 7.7%) studies [35,36]. Convolutional neural networks demonstrated classification accuracies surpassing 90% across various domains [35], while deep neural networks achieved an accuracy of 82% for cognitive impairment and 75% for frailty classification [36].

Internal validation methods, such as k-fold cross-validation, were implemented in 18 studies (69.2%) [24,26,28-36,39-41,43,45,46,48]. In contrast, 6 (23.1%) studies did not explicitly report validation methods [23,25,37,42,44,47], and none included external validation.

### Limitations of Machine Learning in Dementia Research Among People With HIV

The researchers in the included studies acknowledged several methodological challenges. A predominant limitation across studies was small sample size [26,30-32,35-37,39-43,48]. Additionally, study cohorts were largely male, limiting generalizability [23,26,40,43]. Some (4/26, 15.4%) studies highlighted unaddressed confounding variables, such as recreational drug use [37,47], antiretroviral therapy resistance [42], and comorbidities, including hepatitis C virus coinfection [42] and depression [23]. Overlapping variables between HIV-associated neurocognitive disorders and other conditions were also reported [43], along with potential overdiagnosis due to scoring methods such as the Global Deficit Score [33].

The “black box” nature of machine learning models raised concerns about clinical relevance and whether predictive accuracy should outweigh biological plausibility [24,33]. For example, brain connectivity patterns in fMRI data [24] and least absolute shrinkage and selection operator-selected MRI features [33] lacked clear neuropathological explanations, undermining interpretability. Finally, some (3/26, 11.5%) studies did not explicitly acknowledge limitations [28,34,46], potentially omitting critical considerations.

## Discussion

### Principal Findings

This is the first systematic review to examine how machine learning has been applied to dementia-related outcomes in people with HIV, addressing a critical gap in the literature at the intersection of aging, HIV, cognitive decline, and artificial intelligence. Historically, research on cognitive decline in HIV has centered on HIV-associated neurocognitive disorders, including HIV-associated dementia—a form of neurocognitive impairment resulting from the direct effects of HIV on the brain [4,49-56]. This emphasis largely reflected the shorter life expectancy in the early antiretroviral era, when age-related dementias were less prevalent.

We identified 26 studies, most of which focused on neurocognitive impairment related to HIV-associated neurocognitive disorders, with few investigating age-related dementias such as AD and ADRD. This imbalance underscores a major gap in the current literature and highlights the need for future research exploring the full spectrum of cognitive decline among older people with HIV. Although “older adults” is a common term in aging research, many included studies focused on middle-aged populations, reflecting the earlier onset of aging-related concerns in the context of HIV. As people with HIV continue to age, our findings highlight the need for future research to explore the intersection of HIV, aging, and neurodegenerative conditions beyond HIV-associated neurocognitive disorders.

A rigorous study design is essential when applying machine learning techniques in health care research. Most included studies were retrospective or cross-sectional, limiting understanding of disease progression. Because most studies used cross-sectional designs, they were unable to model change over time or to distinguish transient from persistent cognitive impairments. Such designs cannot capture how small errors in measurement or prediction may accumulate and influence dementia trajectories. Future research should use longitudinal designs and temporal modeling approaches to track cognitive changes and evaluate progression risk among older people with HIV.

In addition, retrospective and cross-sectional study designs restrict the ability of current machine learning models to account for real-world contextual factors such as environmental influences, day-to-day variability in cognitive performance, social interactions, and cultural factors that may affect how dementia symptoms manifest or are reported in people with HIV [57,58]. Without longitudinal or ecologically valid data, current machine learning models cannot fully capture the dynamic and multidimensional nature of cognitive changes over time [59,60]. Moreover, most studies did not incorporate contextual or social determinants of health, such as social support, treatment adherence, living conditions, or daily activity patterns, even though these factors are known to influence cognitive trajectories and dementia risk among older people with HIV. Integrating such variables into future machine learning frameworks could enhance the ecological validity and predictive performance of dementia models [61,62]. Longitudinal datasets are also critical for developing models that predict dementia onset or progression [63], as they enable temporal analyses and improve generalizability. Leveraging multimodal data, such as MRI and fMRI, laboratory tests, and neuropsychological assessments across time, can provide a comprehensive view of brain health and facilitate early detection of dementia among older people with HIV.

Integrating biomarker and genetic data with clinical and neuroimaging features offers promise for enhancing prediction of dementia outcomes [64-67]. Of the studies included in this systematic review, only 1 used machine learning to identify key genetic features predictive of HIV-associated neurocognitive disorder status and proposed a framework for biomarker development [48]. Future research should integrate biomarker and genomic information to improve predictive performance and support clinical applications.

Supervised learning methods, including SVMs, logistic regression, and random forests, were the most frequently used and generally demonstrated strong predictive performance across studies. However, despite their high accuracy, supervised machine learning models often require large datasets and substantial computing resources that may limit their scalability and generalizability, particularly in resource-constrained settings or smaller research cohorts [68]. Unsupervised and semisupervised methods (eg, k-means clustering and mutual connectivity analysis) were less common but useful for identifying subgroups and latent cognitive profiles (eg, brain connectivity profiles) [34]. Deep learning methods, although applied in only a few studies, showed promising performance in classifying cognitive impairment and related outcomes. No studies used reinforcement learning, representing an opportunity for future work. As datasets grow in size and complexity, advanced approaches such as deep and reinforcement learning could enhance early detection and personalized risk assessment for dementia in people with HIV [69]. Lessons from these machine learning–based dementia studies may also inform broader efforts to develop scalable, multimodal prediction tools for older populations with complex comorbidities. These approaches can extend beyond HIV-specific contexts to other aging populations facing overlapping challenges in cognitive health and care delivery.

A key limitation identified in this review was the lack of external validation across studies. Although most studies used internal validation methods (eg, k-fold cross-validation), few tested models on external datasets, limiting generalizability and real-world applicability [70]. Future research should prioritize external validation to enhance reproducibility and clinical relevance of machine learning–based dementia prediction in people with HIV.

### Limitations

Our systematic review has several limitations. First, although a comprehensive search strategy was used across multiple databases, it is possible that relevant studies were missed due to publication bias or indexing issues (eg, the limited timeframe, exclusion of non-English publications, or exclusion of gray literature). Second, despite the systematic screening and review process conducted independently by multiple reviewers, the subjective nature of eligibility assessment might introduce potential reviewer bias. To mitigate this, we assessed interrater reliability using the Cohen κ and achieved strong agreement. We also did not perform a formal study quality appraisal; rather, we reported the methodological limitations noted by individual studies. While this approach aligns with rapid review guidance [12-14], it limits formal assessment of study quality. Several included studies relied on self-reported measures, such as subjective memory complaints, which are inherently susceptible to recall bias and response error. These sources of measurement bias were not explicitly addressed or adjusted for in the reviewed studies, limiting the interpretability of self-reported cognitive outcomes. Finally, while our review aimed to categorize and evaluate machine learning techniques used for dementia-related outcomes in people with HIV, the rapid advancements in machine learning may mean that recently published or ongoing studies were not captured at the time of our search; thus, continuous updating is needed in future systematic reviews.

### Conclusions

This review examined how machine learning methods have been applied in dementia research for people with HIV, summarizing the techniques used, their strengths and limitations, and practical implications for future research. Despite promising predictive performance, most studies used supervised machine learning methods and lacked external validation, which limits their generalizability. Future dementia research in HIV could benefit from the adoption of advanced machine learning methods. The dominance of studies focused on HIV-associated neurocognitive disorders, with little attention to age-related neurodegenerative diseases such as AD and ADRD, underscores a critical gap in literature. As the population of people with HIV ages, there is a need to expand longitudinal cohort studies using large-scale real-world data that integrate multimodal information from multiple sources, including clinical covariates, neuroimaging, cognitive assessments, and genetic data, to capture disease progression, identify early biomarkers, and enable personalized risk assessment.

Future machine learning research in this area should ensure methodological rigor, the inclusion of diverse data sources, and external validation to enhance clinical applicability and ultimately improve dementia-related outcomes in older people with HIV. By highlighting current trends and research gaps, this review provides a foundation for advancing machine learning–driven cognitive health research to improve early detection and management of all-cause dementia in older people with HIV. These insights may also inform broader efforts to enhance neurodegenerative outcomes in aging populations through informatics and digital health tools.

## Supplementary material

10.2196/80433 Multimedia Appendix 1 Search strategies.

10.2196/80433 Multimedia Appendix 2 Study characteristics.

10.2196/80433 Multimedia Appendix 3 Machine learning application.

10.2196/80433 Checklist 1 PRISMA checklist.

## Abbreviations

AD: Alzheimer disease
ADRD: Alzheimer disease–related dementias
AUC: area under the curve
fMRI: functional magnetic resonance imaging
MeSH: Medical Subject Headings
MRI: magnetic resonance imaging
SVM: support vector machine
