# Supplementary material for: Applications of Machine Learning for Cognitive Health in Older Individuals With HIV: Rapid Systematic Review
Source: JMIR Aging. 2025 Dec 31;8:e80433. doi: 10.2196/80433 (PMC12755898; doi:10.2196/80433)
Supplement: Multimedia Appendix 1 [file aging-v8-e80433-s001.docx]

**Supplementary File 1. Search Strategies**

**Database: MEDLINE (via PubMed)**

("HIV"[MeSH] OR "HIV Infections"[Mesh] OR "HIV infect*"[tiab] OR "HIV patient*"[tiab] OR "HIV positive patient*"[tiab] OR "HIV sufferer*"[tiab] OR "HIV victim*"[tiab] OR "HIV"[tiab] OR "HIV+"[tiab] OR "HIV/AIDS"[tiab] OR "human immunodeficiency virus"[tiab] OR "PLWH*"[tiab] OR "patient* living with HIV"[tiab] OR "people living with HIV"[tiab] OR "person* living with HIV"[tiab] OR "acquired immunodeficiency syndrome*"[tiab] OR "person* living with AIDS"[tiab] OR "people living with AIDS"[tiab] OR "patient* living with AIDS"[tiab] OR "PLWA"[tiab] OR "women living with AIDS"[tiab] OR "WLHIV"[tiab] OR "AIDS virus*"[tiab])

AND

("Dementia"[MeSH] OR "Dementia*"[tiab] OR "Alzheimer Disease"[MeSH] OR "Alzheimer*"[tiab] OR "Vascular Cognitive Disorder"[tiab] OR "Lewy Body Disease"[tiab] OR "HIV-associated neurocognitive disorder"[tiab] OR "HIV encephalopathy"[tiab] OR "HIV cognitive disorder"[tiab])

AND

("Artificial Intelligence"[MeSH] OR "Machine Learning"[tiab] OR "Artificial Intelligence"[tiab] OR "AI"[tiab] OR "Deep Learning"[tiab] OR "Neural Network*"[tiab] OR "Reinforcement Learning"[tiab] OR "Natural Language Processing"[tiab] OR "Computer Vision" [tiab] OR "Linear Regression"[tiab] OR "Logistic Regression"[tiab] OR "Decision Trees"[tiab] OR "Random Forest"[tiab] OR "Support Vector Machines"[tiab] OR "SVM"[tiab] OR "K-Nearest Neighbors"[tiab] OR "KNN"[tiab] OR "Naive Bayes"[tiab] OR "K-Means Clustering"[tiab] OR "Principal Component Analysis"[tiab] OR "Linear Models"[tiab] OR "Logistic Models"[tiab] OR "Ensemble Methods"[tiab] OR "Tree-Based Methods"[tiab] OR "Kernel Machines"[tiab] OR "Nearest Neighbor Algorithm"[tiab] OR "Bayesian Methods"[tiab] OR "Clustering Algorithms"[tiab] OR "Dimensionality Reduction"[tiab] OR "Feature Extraction"[tiab] OR "Multilayered Perceptrons"[tiab] OR "Gated Recurrent Units"[tiab] OR "GRU"[tiab] OR "Transformer"[tiab] OR "Generative Adversarial Networks"[tiab] OR "GAN"[tiab] OR "Autoencoders"[tiab] OR "Deep Belief Networks"[tiab] OR "DBN"[tiab] OR "Residual Networks"[tiab] OR "ResNet"[tiab] OR "BERT"[tiab] OR "Bidirectional Encoder Representations from Transformers"[tiab] OR "GPT"[tiab] OR "ChatGPT"[tiab] OR "LLaMA"[tiab] OR "Gemini"[tiab] OR "Generative Pre-trained Transformer"[tiab] OR "Attention Mechanism"[tiab] OR "Encoder-Decoder Models"[tiab] OR "Deep Learning Frameworks"[tiab] OR "T5"[tiab] OR "Text-to-Text Transfer Transformer"[tiab] OR "RoBERTa"[tiab] OR "CLIP"[tiab] OR "Contrastive Language-Image Pre-training"[tiab] OR "DALL-E"[tiab] OR "Large Model"[tiab] OR "Large Language Model"[tiab] OR "LLM"[tiab] OR "Large Vision Model"[tiab])

**Database: Embase (via Elsevier.com)**

('human immunodeficiency virus'/exp OR 'human immunodeficiency virus infection'/exp OR 'human immunodeficiency virus infected patient'/exp OR 'aids patient'/exp OR 'hiv infect*':ti,ab OR 'hiv patient*':ti,ab OR 'hiv positive patient*':ti,ab OR 'hiv sufferer*':ti,ab OR 'hiv victim*':ti,ab OR 'hiv':ti,ab OR 'hiv+':ti,ab OR 'hiv/aids':ti,ab OR 'human immunodeficiency virus':ti,ab OR 'plwh*':ti,ab OR 'patient* living with hiv':ti,ab OR 'people living with hiv':ti,ab OR 'person* living with hiv':ti,ab OR 'acquired immunodeficiency syndrome*':ti,ab OR 'person* living with aids':ti,ab OR 'people living with aids':ti,ab OR 'patient* living with aids':ti,ab OR 'plwa':ti,ab OR 'women living with aids':ti,ab OR 'wlhiv':ti,ab OR 'aids virus*':ti,ab)

AND

('dementia'/exp OR dementia*:ti,ab OR 'Alzheimer*':ti,ab OR 'Vascular Cognitive Disorder':ti,ab OR 'Lewy Body Disease':ti,ab OR 'HIV-associated neurocognitive disorder':ti,ab OR 'HIV encephalopathy':ti,ab OR 'HIV cognitive disorder':ti,ab)

AND

('artificial intelligence'/exp OR 'machine learning'/exp OR 'machine learning':ti,ab OR 'artificial intelligence':ti,ab OR 'ai':ti,ab OR 'deep learning':ti,ab OR 'neural network*':ti,ab OR 'reinforcement learning':ti,ab OR 'natural language processing':ti,ab OR 'computer vision':ti,ab OR 'linear regression':ti,ab OR 'logistic regression':ti,ab OR 'decision trees':ti,ab OR 'random forest':ti,ab OR 'support vector machines':ti,ab OR 'svm':ti,ab OR 'k-nearest neighbors':ti,ab OR 'knn':ti,ab OR 'naive bayes':ti,ab OR 'k-means clustering':ti,ab OR 'principal component analysis':ti,ab OR 'linear models':ti,ab OR 'logistic models':ti,ab OR 'ensemble methods':ti,ab OR 'tree-based methods':ti,ab OR 'kernel machines':ti,ab OR 'nearest neighbor algorithm':ti,ab OR 'bayesian methods':ti,ab OR 'clustering algorithms':ti,ab OR 'dimensionality reduction':ti,ab OR 'feature extraction':ti,ab OR 'multilayered perceptrons':ti,ab OR 'gated recurrent units':ti,ab OR 'gru':ti,ab OR 'transformer':ti,ab OR 'generative adversarial networks':ti,ab OR 'gan':ti,ab OR 'autoencoders':ti,ab OR 'deep belief networks':ti,ab OR 'dbn':ti,ab OR 'residual networks':ti,ab OR 'resnet':ti,ab OR 'bert':ti,ab OR 'bidirectional encoder representations from transformers':ti,ab OR 'gpt':ti,ab OR 'chatgpt':ti,ab OR 'llama':ti,ab OR 'gemini':ti,ab OR 'generative pre-trained transformer':ti,ab OR 'attention mechanism':ti,ab OR 'encoder-decoder models':ti,ab OR 'deep learning frameworks':ti,ab OR 't5':ti,ab OR 'text-to-text transfer transformer':ti,ab OR 'roberta':ti,ab OR 'clip':ti,ab OR 'contrastive language-image pre-training':ti,ab OR 'dall-e':ti,ab OR 'large model':ti,ab OR 'large language model':ti,ab OR 'llm':ti,ab OR 'large vision model':ti,ab)

**Database: CINAHL (via EBSCOhost)**

((MH "Human Immunodeficiency Virus+") OR (MH "HIV Infections+") OR (MH "HIV-Positive Persons+") OR "HIV infect*" OR "HIV patient*" OR "HIV positive patient*" OR "HIV sufferer*" OR "HIV victim*" OR “HIV” OR “HIV+” OR “HIV/AIDS” OR “human immunodeficiency virus” OR PLWH* "patient* living with HIV" OR "people living with HIV" OR "person* living with HIV" OR "acquired immunodeficiency syndrome*" OR “person* living with AIDS” OR “people living with AIDS” OR “patient* living with AIDS” OR “PLWA” OR “women living with AIDS” OR “WLHIV” “AIDS virus*”)

AND

((MH "Dementia+") OR Dementia* OR "Alzheimer Disease"[MeSH] OR Alzheimer* OR "Vascular Cognitive Disorder" OR "Lewy Body Disease" OR "HIV-associated neurocognitive disorder" OR "HIV encephalopathy" OR "HIV cognitive disorder")

AND

((MH "Artificial Intelligence+") OR "Machine Learning" OR "Artificial Intelligence" OR "AI" OR "Deep Learning" OR "Neural Network*" OR "Reinforcement Learning" OR "Natural Language Processing" OR "Computer Vision" OR "Linear Regression" OR "Logistic Regression" OR "Decision Trees" OR "Random Forest" OR "Support Vector Machines" OR "SVM" OR "K-Nearest Neighbors" OR "KNN" OR "Naive Bayes" OR "K-Means Clustering" OR "Principal Component Analysis" OR "Linear Models" OR "Logistic Models" OR "Ensemble Methods" OR "Tree-Based Methods" OR "Kernel Machines" OR "Nearest Neighbor Algorithm" OR "Bayesian Methods" OR "Clustering Algorithms" OR "Dimensionality Reduction" OR "Feature Extraction" OR "Multilayered Perceptrons" OR "Gated Recurrent Units" OR "GRU" OR "Transformer" OR "Generative Adversarial Networks" OR "GAN" OR "Autoencoders" OR "Deep Belief Networks" OR "DBN" OR "Residual Networks" OR "ResNet" OR "BERT" OR "Bidirectional Encoder Representations from Transformers" OR "GPT" OR "ChatGPT" OR "LLaMA" OR "Gemini" OR "Generative Pre-trained Transformer" OR "Attention Mechanism" OR "Encoder-Decoder Models" OR "Deep Learning Frameworks" OR "T5" OR "Text-to-Text Transfer Transformer" OR "RoBERTa" OR "CLIP" OR "Contrastive Language-Image Pre-training" OR "DALL-E" OR "Large Model" OR "Large Language Model" OR "LLM" OR "Large Vision Model")
