# Supplementary material for: Applications of Machine Learning for Cognitive Health in Older Individuals With HIV: Rapid Systematic Review
Source: JMIR Aging. 2025 Dec 31;8:e80433. doi: 10.2196/80433 (PMC12755898; doi:10.2196/80433)
Supplement: Multimedia Appendix 2 [file aging-v8-e80433-s002.docx]

**Supplementary File 2. Study Characteristics**

| Author (Publication year) | Location of study | Study objectives | Study design | Study sample | Data sources | Data period | Type of dementia |
| --- | --- | --- | --- | --- | --- | --- | --- |
| Amusan (2020)^1^ | Canada | To examine the association between cumulative antiretroviral therapy (ART) exposure and neurocognitive function | Cohort study | Total sample (n=343)   - Participant group 1 (n=32) - Mean age: 46.8y - Male: 92.4% - Caucasian: 95.5% - Participant group 2 (n=152) - Mean age: 50y - Male: 89.5% - Caucasian: 88.8% - Participant group 3 (n=59) - Mean age: 52y - Male: 89.8% - Caucasian: 88.1% | Neuropsychological test battery | 2020 | Neurocognitive impairment (NCI) |
| Anderson (2018)^2^ | United States | To predict cognitive decline and death among human immunodeficiency virus (HIV) positive (+) patients with increased intraindividual variability | Not reported | Total sample (n=708)   - Age (y) - Baseline: 21-81 - Mean (±SD*): 43.9 (±8.84) - Gender - Male: 78.5% - Education (y) - Mean (±SD): 12.6 (±2.82) - Race/Ethnic - African American: 31.2% - Hispanic: 24.0% - Non-Hispanic Caucasian: 41.3% - Baseline neurocognitive status - 33.1% neurocognitively normal - 20.1% met criteria for asymptomatic neurocognitive impairment (ANI) - 30.9% met criteria for mild neurocognitive disorder (MND) - 16% met criteria for HIV-associated dementia (HAD) | Neurocognitive testing | 1999-2013 | HIV-associated neurocognitive disorder (HAND) |
| Chockanathan (2019)^3^ | United States | To classify individuals as HIV+ or HIV negative (−) and to predict overall cognitive performance | Not reported | Total sample (n=22)   - HIV+ (n=9) - Mean age (±SD; y): 52±10 - 2 females - HIV- (n=13) cognitively normal - Mean age (±SD; y): 41±16 - 6 females | Neuroimaging data: Functional magnetic resonance imaging  (fMRI) | Not explicitly mentioned | HAND |
| DSouza (2017)^4^ | United States | To evaluate the applicability of large-scale Granger Causality (lsGC) analysis for classifying HAND | Not reported | Total sample (n=29)   - HAND (n=14) - Age: 32-53y - Healthy controls (n=15) - Did not report any other demographics including gender or other comorbidities | fMRI data acquired at a single institution | Not explicitly mentioned | HAND |
| DSouza (2017)^5^ | United States | To distinguish between healthy individuals and those with HAND using resting-state fMRI | Not reported | Total sample (n=29)   - HAND (n=14) - Age: 32–53y - Healthy Controls (n=15) - Did not report any other demographics including gender or other comorbidities | Resting-state fMRI scans | Not explicitly mentioned | HAND |
| Finkelstein (2021)^6^ | United States | To evaluate white matter structural integrity in HIV+ individuals using fixel-based analysis and free water corrected diffusion tensor imaging (DTI) and examine correlations with cognitive performance | Not reported | Total sample (n=94)   - Age (Mean±SE^): - HIV+: 34.05 (±2.01) - HIV–: 37.13 (±1.65) - Gender (n, %) - Female: HIV+: (2, 4.8%), HIV–: (26, 50%). - Male: HIV+ :(40, 95.2%), HIV– :(26, 50%). - Ethnicity (n, %) - Hispanic or Latino: HIV+: (2, 4.8%), HIV–: (3, 5.8%). - Not Hispanic or Latino: HIV+: (40, 95.2%), HIV–: (49, 94.2%). - Race (n, %) - Caucasian: HIV+: (20, 47.6%), HIV–: (43, 82.7%). - Black: HIV+: (21, 50%), HIV–: (5, 9.6%). - Other: HIV+: (1, 2.4%), HIV–: (2, 3.8%). - Education Levels (n, %) - Less than 12 years: HIV+: (2, 4.8%), HIV–: (2, 3.8%). - High school graduates (12 years): HIV+: (11, 26%), HIV–: (4, 7.7%). | Magnetic resonance imaging (MRI), blood markers: neurofilaments light (NfL), Tau, and cognitive assessments collected at a single academic medical center. | Not reported | HAND |
| Fu (2022)^7^ | China | To explore the value of machine learning in evaluating gray matter (GM) volume changes in HIV+ patients for early diagnosis of HAND | Not reported | Total sample (n=41)   - Age (Mean±SE) - HIV+: 42.48±13.03 - HIV-: 39±13.02 - Gender - HIV+: Male:14, Female:13 - HIV-: Male:8, Female:6 | MRI data collected at a single academic medical center | September 2017 to January 2019 | HAND |
| Gomez (2019)^8^ | Canada | To identify neurocognitive profiles in ART-treated HIV+ individuals and determine demographic, clinical, and behavioral predictors of impairment | Not reported | Total sample (n=381)   - Age - mean age±SD: 47.3±11.1 - Gender: - Male:88% of the total population. - Place of Origin: - North America: 71.1%. - Cognitive Reserve: - High Cognitive Reserve: 52.4% | Clinical and neurocognitive data were collected from HIV cohorts who active treatment at a universal healthcare clinic | 1999 to 2008 | HAND |
| Levine (2017)^9^ | United States | To characterize effort in the multicenter AIDS cohort study (MACS) to evaluate the potential impact of suboptimal effort on inflated prevalence estimates and fluctuating severity HAND | Cohort study | Total sample (n=57)   - Age: - Mean age±SD: 51±16 - Education (mean year±SD): 14.8 years±2.7 - Two-thirds (66.7%) were HIV+, 58% reported suboptimal effort at baseline. - Race: - White: 65% | Neuroimaging, substance use questionnaire, center for epidemiologic studies depression scale (CES-D), memory self-rating | Not Reported | HAND |
| Luckett (2019)^10^ | United States | To utilize deep neural networks with cerebral blood flow features to classify cognitive impairment (CI) and frailty in people living with HIV (PLWH) | Not reported | Total sample (n=125)   - Age - Years±SD: 51.4±11.4 - Sex: - Male: 66% - Education - Years±SD: 13.7±2.8 - Race: - African American: 53% - Duration of infection - Years±SD: 15.6±8.8 | Neuropsychological tests, frailty assessments, and arterial spin labeling MRI to measure cerebral blood flow collected at a single academic medical center. | Not reported | HAND |
| Martinez- Banfi (2021)^11^ | Colombia | To evaluate the performance of a recently proposed short protocol for detecting  HAND | Cross-sectional | Total sample (n=120)   - Age: - Mean Age±SD: 36.07±10.98 - Education: - Years±SD: 9.02±2.65 - Gender: - Female: 74 - Male: 46 - Sexual Orientation: - Heterosexual: 111 - Homosexual: 5 - Bisexual: 4 - Hand Preference: - Left: 6 - Right: 114 | The mini mental status exam (MMSE) and neuropsychological instruments | Not reported | HAND |
| Narvid (2018)^12^ | United States | To evaluate the specific regional pattern of cerebral blood flow (CBF) abnormalities in older HIV virally suppressed participants using quantitative whole-brain arterial spin-labeling (ASL) | Not reported | Total sample (n=51)   - HIV+:19 - Age (mean years±SD): HIV+: 66.31±3.09 - Healthy controls: - Age (mean years±SD): 69.28±60.74 - Mild cognitive (MC): - Age (mean years±SD): 65.83±4.19 - Years of Education: - HIV+:16.74 - Healthy controls: 16.38±2.69 - MC: 16.47±2.93 - Sex: - HIV+: Male:18, Female: 1 - Healthy controls: Male:13, Female:0 - MC: Male: 19, Female: 0 | Imaging and clinical data collected from a single medical center | Not reported | HAND |
| Ogishi (2018)^13^ | Japan | To predict HAND using machine learning with genetic features derived from the HIV envelope gp120 glycoprotein | Not reported | - 2349 C2V3C3 sequences from 9 studies involving 85 specimens from 43 patients | Los Alamos HIV sequence database and literature-derived datasets | Not reported | HAND |
| Pinheiro (2021)^14^ | Brazil | To identify the algorithm with the best performance capable of revealing the most relevant characteristics in the high risk of developing dementia in people with HIV/AIDS based on data mining | Not reported | Total sample: (n=270)   - Age group - 50-55 (n=133) - 56-60 (n=75) - 61-70 (n=52) - >70 (n=10) - Gender: - Female: 91 - Male: 179 - Marital status - Not married (n=114) - Married (n=71) - Widowed (n=43) - Divorced (n=42) - Education - Incomplete fundamental (n=89) - Completed elementary school (n=46) - High school (n=61) - Higher (n=29) - Illiterate (n=45) | Sociodemographic profile, history of infectious disease, drug adherence, neurological assessment, psychosomatic changes, daily activities | January to April 2019 | HAND |
| Pulliam (2020)^15^ | United States | To predict HAND using plasma neuronal extracellular vesicle (nEV) proteins and clinical data with machine learning | Not reported | Total sample (n=60)   - HIV+: - Female:38, Male:22 - Neuropsychological impairment (NPI) (n=40) - Neuropsychologically normal (NPN) (n=20). - Age (mean age±SD) - NPN: 46.1±8.4 - NPI: 47.8±10.4 - Education (mean years±SD) - NPN: 13.6±2.6 - NPI: 12.4±2.9 - Sex: - NPN: Women:14, Men:6 - NPI: Women:14, Men:16 - Ethnicity: - NPN: - Black:2, - Caucasian:11 - Hispanic/ other:7 - NPI: - Asian:1 - Black:11 - Caucasian:20 - Hispanic/other:8 | Plasma samples from the National NeuroAIDS Tissue Consortium and HIV Neurobehavioral Research Program | Not reported | HAND |
| Qi (2023)^16^ | China | To evaluate the utility of radiomic analysis combined with DTI in identifying ANI in PLWH | Not reported | Total sample (n=208)   - Healthy Controls: 70 participants. - HIV with Intact Cognition (IC): 68 participants. - HIV with asymptomatic neurocognitive impairment (ANI): 70 participants. - Age (Median [IQR]): - Healthy controls: 32.0 [29.5, 37.0]. - IC: 33.5 [29.3, 37.8]. - ANI: 33.5 [29.0, 37.0]. - Education Level (Years, Median [IQR]): - Healthy controls: 16.0 [14.0, 18.0]. - IC: 16.0 [15.0, 16.0]. - ANI: 15.0 [14.3, 16.0]. | Imaging and clinical data collected from a single medical center | November 2020 to August 2021 | ANI, an early stage of HAND |
| Qi (2021)^17^ | China | To investigate differences in putamen volume and radiomic features between ANI and pre-clinical ANI in HIV patients and explore correlations with cognitive function | Not reported | Total sample (n=90)   - Age (mean year±SD): - ANI (30.54±5.183) - Pre-clinical ANI: (32.79±7.176) - Duration of Infection (months): - ANI: Median 19 (IQR: 9–40) - Pre-clinical ANI: Median 24 (IQR: 9–48) - Duration of Treatment (months): - ANI: Median 16.0 (IQR: 6.3–24.8). - Pre-clinical ANI: Median 20.0 (IQR: 9.8–47.0). | MRI data and neuropsychological test results | 2015 to June 2019 | HAND (ANI and pre-clinical ANI) |
| Salahuddin (2020)^18^ | Ethiopia | To assess the prevalence and predictors of neurocognitive impairment, HIV patients were assessed in a behavioral battery of recall memory task, motor function task, and psychomotor tasks using the  international HIV dementia scale (IHDS) | Cross-sectional | Total sample (n=244)   - Age groups: - Up to 40: (n=207) - 40 and above: (n=37) - Gender: - Male: 89, - Female: 155 - Education: - Primary education and lower: (n=169) - Secondary education and higher: (n=75) - Marital status: - Single: (n=75) - Married: (n=169) - Income: - Very low: (n=57) - Low: (n=99) - Average: (n=38) - Above average: (n=29) - High: (n=21) - Residence: - Urban: (n=210) - Rural: (n=34) - Recreational drugs: - No: (n=183) - Yes: (n=61) | Clinical data and neuropsychological tests | February 2018 to April 2018 | HAND |
| Tu (2020)^19^ | Canada | To determine the prevalence and subtypes of neurocognitive impairment (NCI) in HIV-infected patients and identify associated variables using machine learning models | Not reported | Total sample (n=370)   - Age (years): - Neurocognitively normal (NN): (47.6), - HAND: (47.4), - Neurocognitively impaired-other disorders (NCI-OD): (45.5). - Sex (female): - NN: 14.6%, - HAND: 10.3%, - NCI-OD: 6.5%. - Education (years): - NN: (14.0) - HAND: (14.0) - NCI-OD: (13.2) - Employment: - NN: (69.5%) - HAND: (62.8%) - NCI-OD: (56.5%) - Substance Use: - Cigarette Use: - NN: (22.4%) - HAND: (17.9%) - NCI-OD: (34.8%) - Crack/Cocaine Use: - NN: (8.9%) - HAND: (5.1%) - NCI-OD: (6.5%). - Alcohol (binge): - NN: (4.5%) - HAND: (1.3%) - NCI-OD: (10.9%) - Marijuana: - NN: (28.9%) - HAND: (20.5%) - NCI-OD: (37.0%) - Polypharmacy: - Higher in NCI-OD (5.3 medications) compared to HAND (4.1) and NN (4.2). | Clinical data, neuropsychological tests, and demographic data from the southern Alberta HIV clinic database | Not specified; data includes long-term follow-up with universal health care. | HAND and NCI-OD. |
| Underwood (2018)^20^ | United States | To use multivariate pattern analysis of volumetric neuroimaging data to predict cognitive function in virally suppressed HIV-positive individuals | Not reported | Total sample (n=139)   - Age: - Median 44 years (IQR: 44–50) - Gender: - Male:110(79.1%) - Ethnicity: - White: 64 (46.0%) - Black: 61 (43.9%) - Hispanic: 13 (9.4%) - Other: 1 (0.7%) - Years of Education: - Median 13.0 years (IQR: 12.0–15.0). - Weight: - Median 77.7 kg (IQR: 70.5–87.8). - Duration of HIV Infection: - Median 12.2 years (IQR: 6.3–15.8). - Current CD4+ Count: - Median 540 cells/mL (IQR: 353–698). - Duration of Antiretroviral Therapy (ART): - Median 6.3 years (IQR: 2.6–9.4). | T1-weighted MRI neuroimaging data and cognitive assessments collected from 6 participating university centers | September 2003 and ended in August 2007 (Baseline and longitudinal cognitive data (mean 3.5 visits over 6 months) | HAND |
| Xu (2021)^21^ | United States | To predict NCI among people with HIV using clinical data and multimodal MRI features | Not reported) | Total sample (n=101)   - Demographics characteristics - Age (mean age±SD): - Neurocognitive impairment negative (NCI-): 42.1±9.7. - Neurocognitive impairment positive (NCI+): 40.9±9.5. - Male: - NCI−: 28 (66.7%). - NCI+: 46 (78.0%) - African American Race: - NCI−: 29 (69.1%). - NCI+: 53 (89.8%). - Current Substance Use: - Daily Cigarette Smoking: - NCI−: 20 (47.6%). - NCI+: 34 (57.6%). - Alcohol Use: - NCI−: 38 (90.5%). - NCI+: 43 (72.9%). - Cocaine Use: - NCI−: 14 (33.3%). - NCI+: 17 (28.8%). - Marijuana Use: - NCI−: 17 (40.5%). - NCI+: 24 (40.7%). - HIV Characteristics: - Years Since HIV Diagnosis: - NCI−: Median 8.4 (IQR 5.2, 15.5). - NCI+: Median 8.0 (IQR 3.7, 17.6). - Recent CD4+ T Cell Count (cells/mL): - NCI−: Median 711 (IQR 480, 857). - NCI+: Median 513 (IQR 300, 860). - Nadir CD4+ T Cell Count (cells/mL): - NCI−: Median 305 (IQR 105, 500). - NCI+: Median 228 (IQR 100, 386). - Recent Viral Load < 50 copies/mL: - NCI−: 31 (75.6%). - NCI+: 44 (75.9%). | Clinical data (demographics, substance use, laboratory tests) and MRI (gray matter volume, diffusion-weighted imaging for white matter integrity) | Not explicitly stated (even in the previous studies cited) | HAND, NCI |
| Yang (2021)^22^ | United States | To address these gaps by (1) identifying comorbidity clusters; (2) examining the patterns of comorbidity in terms of concurrence of comorbidity clusters as well as the temporal trend in the prevalence of diﬀerent patterns of concurrence across a long follow up period | Cohort study | Total sample: (n=8490)   - Age: - At least 45 years old (n=2,290) - Sex - Male: (n= 6,367) - Female: (n=2123) - Race - Black: (n=6,004) - White: (n=1,872) - Hispanic/others: (n=614) | Electronic health record (EHR) data | January 2005- December 2016 | Dementia (not explicitly explained) |
| Zhan (2024)^23^ | China | To investigate the altered brain metabolites in ANI patients comparing to PLWH with normal cognition, and further explore their relationships with neuropsychological (NP) deficits | Cross-sectional study | Total sample: (n=80)   - HIV ANI male (n=29) - HIV normal male (n=47) - Age (mean age±SD) - HIV ANI 39.06±12.715 - HIV normal 35.53±8.026 - Education years - HIV ANI 13.71±2.795 - HIV normal 14.20±2.458 | MRI imaging and spectroscopy | 2015-2019 | ANI |
| Zhang (2022)^24^ | United States | To develop and validate a multi-label, multi-domain model to classify brain MRI data based on HIV status and cognitive impairment (e.g., HAND) | Not reported | Total sample (n=1449)   - Memory and aging center at University of California San Francisco (UCSF) dataset: - Control (n=156) - HIV-only (n=37) - Cognitive Impairment (CI)-only (335) - HAND (n=145) - Age (mean age±SD) - Control 70.1±5.9 - HIV-only 64.9±3.8 - CI-only 67.3±7.3 - HAND 64.1±5.0 - Sex (Male, Female) - Control (147 M, 10F) - HIV-only (36M, 1F) - CI-only (165M, 170F) - HAND (136M, 9F) - Stanford Research Institute (SRI) dataset - Control (n=75), - HIV-only (n=75) - Age (mean age±SD) - Control 51.7±10.6 - HIV-only 51.0±12.5 - Sex (Male, Female) - Control (53M, 22F) - HIV-only (53M, 22F) - Alzheimer’s Disease Neuroimaging Initiative (ADNI) dataset: - Control (n=229) - CI-only (n=397) - Age (mean age±SD) - Control 75.9±5.0 - CI-only 74.8±7.4 - Sex (Male/ Female) - Control (119M, 110F) - CI-only (254 M, 143 F) | Brain MRIs, including volumetric measures, from UCSF (HIV-focused cohort), SRI (HIV-focused cohort), and ADNI (Alzheimer’s-focused cohort) | Not explicitly stated (even in the previous studies cited) | HAND, CI |
| Zhang (2016)^25^ | United States | To identify anatomical patterns distinguishing HAND from mild cognitive impairment (MCI) via the following novel study design | Not reported | - UCSF HIV over 60 cohort study (UHES) dataset - Age (mean age±SD):   HAND 63.8±3.3; Control 65.1±3.2   - Gender:   HAND: Male 14, Female1; Control: Male 19, Female 2   - ADNI dataset - Gender: - MCI: Male:70, Female:10 - Age (mean age±SD): - MCI: 65.1±3.22 - Siemens: - Gender: - Male 15, Female 3 - Age (mean age±SD): - 65.2±3.2 - GE machines: - Gender: - Male 7, Female 1 - Age (mean age±SD): - 65.6±3.2 | Neuroimaging | Not reported | HAND, MCI |
| Zhu (2019)^26^ | China | To identify symptom clusters and examine demographic and health-related factors  associated with these symptom clusters among PLWH prescribing antiretroviral therapy in China | Cross-sectional | Total sample (n=1116)   - Characteristics: - Age: - Baseline:32- 50y - Gender: - Male: 804, Female: 312 - Race/ethnicity: - Han: 888 (79.57%) - Minority: 228 (20.43%) - Educational level: - Middle school or below: 586 (52.51%) - High school or equivalent: 220 (19.71%) - Post grad or equivalent: 279 (25%) - Master’s or above: 31 (2.78%) - Having religion: 118 (10.57%) | fMRI data acquired at a single institution | Not explicitly mentioned | HAND |

*SD: Standard Deviations; ^SE: Standard Error

**References**

1. Amusan P, Power C, Gill MJ, et al. Lifetime antiretroviral exposure and neurocognitive impairment in HIV. *Journal of NeuroVirology*. 2020;26(5):743-753. doi:10.1007/s13365-020-00870-z

2. Anderson AE, Jones JD, Thaler NS, Kuhn TP, Singer EJ, Hinkin CH. Intraindividual variability in neuropsychological performance predicts cognitive decline and death in HIV. *Neuropsychology*. 2018;32(8):966-972. doi:10.1037/neu0000482

3. Chockanathan U, Dsouza AM, Abidin AZ, Schifitto G, Wismüller A. Automated diagnosis of HIV-associated neurocognitive disorders using large-scale Granger causality analysis of resting-state functional MRI. *Computers in Biology and Medicine*. 2019;106:24-30. doi:10.1016/j.compbiomed.2019.01.006

4. Am DS, Abidin AZ, Leistritz L, Wismüller A. Identifying HIV Associated Neurocognitive Disorder Using Large-Scale Granger Causality Analysis on Resting-State Functional MRI. *Proc SPIE Int Soc Opt Eng*. 2017;10133doi:10.1117/12.2254690

5. Am DS, Abidin AZ, Wismüller A. Investigating Changes in Resting-State Connectivity from Functional MRI Data in Patients with HIV Associated Neurocognitive Disorder Using MCA and Machine Learning. *Proc SPIE Int Soc Opt Eng*. 2017;10137doi:10.1117/12.2254189

6. Finkelstein A, Faiyaz A, Weber MT, et al. Fixel-Based Analysis and Free Water Corrected DTI Evaluation of HIV-Associated Neurocognitive Disorders. *Frontiers in Neurology*. 2021;12doi:10.3389/fneur.2021.725059

7. Fu D, Mo K, Deng W, et al. Application Value of Machine Learning Method in Measuring Gray Matter Volume of AIDS Patients. *Disease Markers*. 2022;2022doi:10.1155/2022/1210002

8. Gomez D, Power C, Gill MJ, Koenig N, Vega R, Fujiwara E. Empiric neurocognitive performance profile discovery and interpretation in HIV infection. *J Neurovirol*. 2019;25(1):72-84. doi:10.1007/s13365-018-0685-6

9. Levine AJ, Martin E, Sacktor N, Munro C, Becker J. Predictors and Impact of Self-Reported Suboptimal Effort on Estimates of Prevalence of HIV-Associated Neurocognitive Disorders. *Journal of Acquired Immune Deficiency Syndromes*. 2017;75(2):203-210. doi:10.1097/QAI.0000000000001371

10. Luckett P, Wisch J, Cooley SA, Ances BM. Deep-learning cerebral blood flow for cognitive-impairment classification in HIV. *Topics in Antiviral Medicine*. 2019;27(SUPPL 1):46s.

11. Martinez-Banfi M, Vélez JI, Mebarak Chams MR, et al. Utility of a short neuropsychological protocol for detecting HIV-associated neurocognitive disorders in patients with asymptomatic HIV-1 infection. *Brain Sciences*. 2021;11(8)doi:10.3390/brainsci11081037

12. Narvid J, McCoy D, Dupont SM, et al. Abnormal cerebral perfusion profile in older adults with HIV-associated neurocognitive disorder: Discriminative power of arterial spin-labeling. *American Journal of Neuroradiology*. 2018;39(12):2211-2217. doi:10.3174/ajnr.A5902

13. Ogishi M, Yotsuyanagi H. Prediction of HIV-associated neurocognitive disorder (HAND) from three genetic features of envelope gp120 glycoprotein. *Retrovirology*. 2018;15(1)doi:10.1186/s12977-018-0401-x

14. Pinheiro L, Pereira MLD, Fernandez MP, Filho FMV, de Abreu W, Pinheiro P. Application of Data Mining Algorithms for Dementia in People with HIV/AIDS. *Comput Math Methods Med*. 2021;2021:4602465. doi:10.1155/2021/4602465

15. Pulliam L, Liston M, Sun B, Narvid J. Using neuronal extracellular vesicles and machine learning to predict cognitive deficits in HIV. *Journal of NeuroVirology*. 2020;26(6):880-887. doi:10.1007/s13365-020-00877-6

16. Qi Y, Wang W, Rao B, et al. Value of Radiomic Analysis Combined With Diffusion Tensor Imaging in Early Diagnosis of HIV-Associated Neurocognitive Disorders. *Journal of Magnetic Resonance Imaging*. 2023;58(6):1882-1891. doi:10.1002/jmri.28741

17. Qi Y, Xu M, Wang W, et al. Early prediction of putamen imaging features in HIV-associated neurocognitive impairment syndrome. *BMC Neurology*. 2021;21(1):1-10. doi:10.1186/s12883-021-02114-x

18. Salahuddin M, Manzar MD, Hassen HY, et al. Prevalence and Predictors of Neurocognitive Impairment in Ethiopian Population Living with HIV. *HIV AIDS (Auckl)*. 2020;12:559-572. doi:10.2147/hiv.S260831

19. Tu W, Chen PA, Koenig N, et al. Machine learning models reveal neurocognitive impairment type and prevalence are associated with distinct variables in HIV/AIDS. *Journal of NeuroVirology*. 2020;26(1):41-51. doi:10.1007/s13365-019-00791-6

20. Underwood J, Cole JH, Leech R, Sharp DJ, Winston A. Multivariate Pattern Analysis of Volumetric Neuroimaging Data and Its Relationship With Cognitive Function in Treated HIV Disease. *J Acquir Immune Defic Syndr*. 2018;78(4):429-436. doi:10.1097/qai.0000000000001687

21. Xu Y, Lin Y, Bell RP, et al. Machine learning prediction of neurocognitive impairment among people with HIV using clinical and multimodal magnetic resonance imaging data. *J Neurovirol*. 2021;27(1):1-11. doi:10.1007/s13365-020-00930-4

22. Yang X, Zhang J, Chen S, Weissman S, Olatosi B, Li X. Comorbidity patterns among people living with HIV: a hierarchical clustering approach through integrated electronic health records data in South Carolina. *AIDS Care*. 2021;33(5):594-606. doi:10.1080/09540121.2020.1844864

23. Zhan Y, Cai DC, Liu Y, et al. Altered metabolism in right basal ganglia associated with asymptomatic neurocognitive impairment in HIV-infected individuals. *Heliyon*. 2024;10(1):e23342. doi:10.1016/j.heliyon.2023.e23342

24. Zhang J, Zhao Q, Adeli E, et al. Multi-label, multi-domain learning identifies compounding effects of HIV and cognitive impairment. *Medical Image Analysis*. 2022;75doi:10.1016/j.media.2021.102246

25. Zhang Y, Kwon D, Esmaeili-Firidouni P, et al. Extracting patterns of morphometry distinguishing HIV associated neurodegeneration from mild cognitive impairment via group cardinality constrained classification. *Human Brain Mapping*. 2016;37(12):4523-4538. doi:10.1002/hbm.23326

26. Zhu Z, Hu Y, Xing W, et al. Identifying Symptom Clusters Among People Living With HIV on Antiretroviral Therapy in China: A Network Analysis. *Journal of Pain and Symptom Management*. 2019;57(3):617-626. doi:10.1016/j.jpainsymman.2018.11.011
