# Supplementary material for: Applications of Machine Learning for Cognitive Health in Older Individuals With HIV: Rapid Systematic Review
Source: JMIR Aging. 2025 Dec 31;8:e80433. doi: 10.2196/80433 (PMC12755898; doi:10.2196/80433)
Supplement: Multimedia Appendix 3 [file aging-v8-e80433-s003.docx]

**Supplementary File 3. Findings of Machine Learning Application**

| Author (Publication year) | Main aim of using machine learning (ML) | Type of ML techniques | Evaluation metrics used for ML models | Key outcomes from ML models | ML-related limitations reported in the article |
| --- | --- | --- | --- | --- | --- |
| Amusan (2020)^1^ | To predict cognitive decline. | Random Forest Analyses. | - High-Performing vs. Impaired Group: - Area under the receiver operating characteristic curve (AUC)=0.846; - *F*₁-score=0.79. - Immediate vs. Impaired Group: - AUC=0.791; - *F*₁-score=0.75. | - Out of a total of 26 tested variables, two random forest analyses identified the most important characteristics of a neurocognitively impaired group (n=59). - Compared with a neurocognitively high-performing group (n=132; *F*₁-score=0.79), they uncovered 13 important risk factors. | Cohorts were mostly born in North America; causality cannot be inferred with this study design; possible that these effects are related to additional treatment factors (e.g., resistance to certain antiretroviral therapy (ART)) or comorbidities (e.g., hepatitis C virus (HCV) coinfection) which remained unexplored due to the limited number of cases in our cohort of neurocognitively impaired individuals. |
| Anderson (2018)^2^ | To examine predictors of dispersion. | Mixed-effects regression model in Model 1 (Predicting cognitive impairment using dispersion), 2 (Predicting cognitive impairment using change in dispersion), and 3 (Predicting death using dispersion). | - Model 1: - Predicting cognitive impairment using dispersion [Log-likelihood=3,546.88; Akaike information criterion (AIC)=7,115.76]. - Model 2: - Predicting cognitive impairment using change in dispersion [Log-likelihood=3,548.69; AIC=7,119.37] - Model 3: - Predicting death using dispersion [AIC=2,021.5; Bayesian information criterion (BIC)=2,077.5; Log-likelihood=1,001.7]. | - Model 1: - Predicting cognitive impairment using dispersion. Dispersion of neurocognitive assessments increased the chance of future cognitive decline (p=.001), as did age (p=.001). - Education was protective against cognitive decline (p=.05), along with male gender (p=.001), baseline mean cognitive functioning (p=.001), and time followed (p=.001). - Model 2: - Predicting cognitive impairment using change in dispersion. Increased change in dispersion between the previous and current visit was significantly predictive of cognitive decline (p=.001), along with time followed (p=.001) and age (p=.001). - Protective factors for cognitive decline included male gender (p=.001), baseline mean cognitive functioning (p=.001), and education (p=.01). - Model 3: - Predicting death using dispersion. Dispersion from the most recent visit was significantly predictive of death after that visit (p=.01). - Additional risk factors for death included time followed (p=.001) and age (p=.001). | This study had a predominantly male sample (78.5%), so these results may not accurately reflect the relationship between dispersion and cognitive decline/death in a female population. |
| Chockanathan (2019)^3^ | To compare the predictive ability of machine learning models trained on graphs derived from three time-series analysis techniques. | Support Vector Machine (SVM). | - AUC for HIV status, coefficient of determination R2 for neuropsychometric (NP) score regression; result cannot be readable. | - SVM classifiers based on large-scale Granger Causality (lsGC)-derived brain graph features significantly outperformed those based on conventional Pearson correlation (PC)-derived features (p<0.05, Bonferroni-corrected). | Since the brain's true connectivity structure is unknown, it is unclear whether the graph properties used by machine learning models to predict disease state and cognitive performance reflect actual differences in information transfer between brain regions or another clinically relevant aspect of the blood-oxygen-level-dependent (BOLD) signal. |
| DSouza (2017)^4^ | To classify HIV associated neurocognitive disorder (HAND) by analyzing brain connectivity patterns. | lsGC and conditional Granger causality (cGc) method for connectivity analysis in pattern recognition; Generalized Matrix Learning Vector Quantization (GMLVQ) for classification. | - Accuracy=87%. - AUC=0.90 for lsGC (better than cGC with: - Accuracy=76%. - AUC=0.74. | - lsGC combined with GMLVQ demonstrated high accuracy and AUC, outperforming traditional cGC methods. | High dimensionality and parameter dependency for cGC; small dataset size for broader generalization. |
| DSouza (2017)^5^ | To identify connectivity changes and classify HAND based on brain functional connectivity patterns. | Combination of unsupervised mutual connectivity analysis (MCA) and supervised (GMLVQ). | - AUC for classification: - MCA with local models (LM)=0.87~0.89. | - MCA-based connectivity analysis outperformed conventional cross-correlation in classifying HAND vs. healthy individuals. - MCA-LM has potential to serve as a diagnostic biomarker for HAND. | Not specified. |
| Finkelstein (2021)^6^ | To classify cognitive impairment in HIV-infected (HIV+) individuals and explore relationships between white matter (WM) degeneration and cognitive performance. | AdaBoost, Random Forest, Naïve Bayes, Linear Discriminant Analysis (LDA). | - AdaBoost achieved the highest precision (0.80), recall (0.77), and *F*₁-score (0.76) for Fixel-based analysis (FBA) metrics. - Other LDA, Random Forest, and Naïve Bayes showed poor performance with a majority of 0.38-0.58. | - FBA metrics better distinguished cognitive impairment in HIV+ individuals compared to free water corrected diffusion tensor imaging (fwcDTI) metrics. - Axonal degeneration in specific fiber tracts. E.g., posterior limb of internal capsule (PLIC), superior corona radiata (SCR) was associated with cognitive performance. | Small sample size, gender imbalance in HIV+ group, dependency of fiber density (FD) metrics on b-values, potential overfitting due to small data size. |
| Fu (2022)^7^ | To predict baseline and longitudinal cognitive function and to investigate deviations in brain age using magnetic resonance imaging (MRI) data. | Multivariate models using SVM, Gaussian Processes Classification (GPC), Kernel Ridge Regression (KRR), and Gaussian Processes Regression (GPR). | - Baseline prediction: - SVM AUC=0.56; - GPC AUC=0.59. - Baseline regression: - KRR R²=0.08; - GPR R²=0.07. - Longitudinal change prediction was poor: - SVM AUC=0.42; - GPC AUC=0.43. | - Multivariate models were superior to linear models for baseline cognitive function but not clinically useful with poor performance. - Brain-predicted age was slightly older than chronological age in HIV+ individuals and associated with comorbidities and prior AIDS. | Small sample size, heterogeneity in cognitive impairment severity, limited predictive power of MRI data alone for mild impairment, and potential scanner-related variability. |
| Gomez (2019)^8^ | To classify neurocognitive profiles and identify factors associated with neurocognitive impairment among HIV + individuals. | Unsupervised learning: latent profile analysis (LPA) and supervised learning random forest analyses (RFA). | - LPA identified three profiles: - Profile 1 (41.7%): Highest neurocognitive performance. - Profile 2 (42.8%): Intermediate neurocognitive performance. - Profile 3 (15.5%): Globally impaired. - RFA (AUC=0.82) identified key predictors for: - Profile 3, including non-North American origin, toxoplasmosis seropositivity, female sex, longer HIV duration, and psychiatric disorders. | - LPA revealed heterogeneity in neurocognitive performance among ART-treated individuals. - RFA highlighted demographic and clinical predictors for the most impaired group (Profile 3). | Lack of HIV-uninfected controls and the limited size of the cohort.  Although established normative adjustments were applied to all tests, reference data accommodating the unique background of the current cohort were unavailable. |
| Levine (2017)^9^ | To determine the predictors of effort level. | Unsupervised learning (K-means clustering) and supervised learning (regression). | - Adjusted - R^2^=0.091; - R^2^ change=0.110; - F change=5.579. - Significance of F change=0.023. | - Predictors of suboptimal effort were self-reported memory impairment, African American race, and cocaine use. - Change in effort between baseline and follow-up correlated with change in HAND severity. | Prevalence estimate: the ending reported here that almost as many human immunodeficiency virus type 2 (HIV2) and HIV+ MACS participants meet criteria for HAND underscores the poor specificity of current diagnostic criteria. |
| Luckett (2019)^10^ | To classify Persons Living with HIV (PLWH) into cognitively impaired or unimpaired groups and further classify frailty status (frail, pre-frail, non-frail). | Deep Neural Networks (DNN) for classification; Relief algorithm for feature importance ranking. | - Cognitive Impairment Classification: - Overall Accuracy=82%; - AUC=0.81; - Learning=85% accuracy; - Memory=86% accuracy; - Executive=86% accuracy; - Frailty Classification=75% accuracy. | - DNN effectively classified cognitive impairment and frailty in PLWH with high accuracy. - Cognitive impairment reflected cortical and subcortical changes, while frailty was primarily subcortical. | Cross-sectional design limits causal interpretations.  Misclassification predominantly occurred in pre-frail group.  Small sample size and need for longitudinal data. |
| Martinez- Banfi (2021)^11^ | To assess the HAND detection performance of our short neuropsychological protocol, and to compare it with the mini-mental state examination (MMSE) and international HIV dementia scale (IHDS) screening tests, we defined HAND status according to three operational criteria: (1) MMSE ≤ 26, (2) IHDS ≤ 10, and (3) both MMSE ≤ 26 and IHDS ≤ 10—the latter determines whether the screening of cognitive impairment associated with both cortical and subcortical is present in human immunodeficiency virus type 1 (HIV-1)infected individuals. | Classification and Regression Tree (CART). | - AUC: - Short Protocol=0.925; - MMSE=0.825; - IHDS=0.867; - MMSE + IHDS=0.900. - Sensitivity (Se): - Short Protocol=0.917; - MMSE=0.883; - IHDS=0.883; - MMSE + IHDS=0.883. - Specificity (Sp): - Short Protocol=0.933; - MMSE=0.817; - IHDS=0.850; - MMSE + IHDS=0.917. - Correct Classification Rate (CCR): - Short Protocol=0.925; - MMSE=0.825; - IHDS=0.876; - MMSE + IHDS=0.900. - Positive Predictive Value (PPV): - Short Protocol=0.932; - MMSE=0.820; - IHDS=0.855; - MMSE + IHDS=0.914. - Negative Predictive Value (NPV): - Short Protocol=   0.918;   - MMSE=0.831; - IHDS=0.879; - MMSE + IHDS= 0.887. - False Discovery Rate (FDR): - Short Protocol=0.068; - MMSE=0.180; - IHDS=0.145; - MMSE + IHDS=0.086. - False-Positive Rate (FPR): - Short Protocol=0.067; - MMSE=0.183; - IHDS= 0.150; - MMSE + IHDS=0.083. - Lift: - Short Protocol=1.864; - MMSE=1.639; - IHDS=1.710; - MMSE + IHDS=1.828. | - Advanced recursive partitioning approach (ARPA)-based HAND predictive model, involving the MMSE criterion, yielded outstanding specificity and correct classification rates, but low-to-average specificity and AUC. - Our results show that our short neuropsychological protocol outperforms other instruments used for the same purpose in the clinical setting, such as the University of California, San Diego (UCSD) Performance-Based Skills Assessment (UPSA-B), which only has an accuracy of 71% when identifying HIV-1 infected individuals with neurocognitive impairment. We also found that when using the IHDS-based operational criterion as a screening tool for HAND, most of the diagnoses were assigned to individuals with HIV-1 Infection. | Only recruited individuals with asymptomatic HIV-1 Infection, which impeded us from testing our ARPA-based predictive models for HAND in individuals with symptomatic HIV or AIDS. |
| Narvid (2018)^12^ | To create a predictive model to differentiate those HIV from healthy controls (respectively) and mild cognitive impairment (MCI) based on cerebral blood flow (CBF), and; To perform casual inference by identifying the key CBF brain regions that are different between the diagnostic groups. | Logistic elastic net regression. | - 1) Prediction of HIV versus healthy controls: (median) - Accuracy=75%; - Sensitivity=87%; - Specificity=57%. - 2) Prediction of HIV versus MCI: - Accuracy=0.73%; - Sensitivity=75%; - Specificity=71%. | - Specific CBF patterns associated with HIV status despite viral suppression—data that should animate further investigations into the pathobiological basis of vascular and cognitive abnormalities in HAND. | The population is almost entirely male, and the sample sizes, especially for the uninfected population, are moderate. |
| Ogishi (2018)^13^ | To develop a predictive biomarker, improve classification accuracy for HAND, and stratify cases into genetic clusters. | Stacked classifiers: SVM, RF, gradient boosting machine (GBM), extreme gradient boosting with tree booster (XGBT) for prediction; k-means clustering for stratification of HAND into subgroups. | - 100% for testing subset - 94% for the entire dataset. | - Identified three key genetic features that predict HAND status with high accuracy, stratified HAND into four genetic clusters, and demonstrated a framework for biomarker development. | As retrospective observational study, no causative link can be definitively established. Small sample size, potential for overfitting, and limited generalizability due to reliance on datasets derived from severe HAND cases. |
| Pinheiro (2021)^14^ | To improve a prediction model. | Logistic regression (LR), decision tree, neural networks, K-nearest neighbors (KNN), random forest (RF). | - No principal component analysis (PCA): Accuracy, precision, recall: - LR=(0.7805/0.7377/ 0.9574); - Decision tree=(0.7317/0.7451/ 0.8085); - Neural network=(0.5732/0.5732/ 1.0); - KNN=(0.6098/0.6415/ 0.7234); - RF=(0.8293/ 0.7797/ 0.9787). | - When considering only 23 characteristics, the precision of the algorithms was 86% in random forest, 56% logistic regression, 68% decision tree, 60% KNN, and 59% neural network. - The random forest algorithm proved to be more eﬀective than the others, obtaining 84% precision and 86% accuracy. | Not explicitly mentioned |
| Pulliam (2020)^15^ | To develop predictive models for identifying cognitive impairment and explore the contributions of clinical and neuronal extracellular vesicle (nEV) biomarkers. | SVM, ensemble KNN, and AdaBoost. | - SVM achieved the best AUC=0.82 (using clinical parameters) and High-mobility group box 1 (HMGB1). - Ensemble KNN=0.8. - AdaBoosting=0.79. | - SVM with clinical parameters and HMGB1 had the best performance; neurofilament light (NFL) added predictive value in other algorithms but not in SVM; p-T181-tau did not contribute to HAND prediction. | Small sample size; limited nEV biomarkers; nonlinear relationships between variables. |
| Qi (2023)^16^ | To improve the early diagnosis of asymptomatic neurocognitive impairment (ANI) by combining radiomic signatures with clinical data. | Least absolute shrinkage and selection operator (LASSO)-logistic algorithm. | - AUC for the nomogram: - Training=0.853; - Validation=0.874. | - The combined use of DTI-based radiomics and clinical data significantly improved ANI diagnosis compared to traditional imaging or clinical models alone. | Small sample size focused only on male patients, limited to a single center; potential selection bias. Only DTI imaging data was analyzed. |
| Qi (2021)^17^ | To classify ANI vs. pre-clinical ANI using radiomic features and assess correlations between cognitive function and putamen volume. | SVM with PCA-based feature selection for classification. | - AUC=0.85 ± 0.22. - Sensitivity=63.12% ± 5.51. - Specificity=94.25% ± 3.08. | - Putamen volume was larger in ANI compared to pre-clinical ANI. - Cognitive functions negatively correlated with the putamen volume. - Radiomic features of the putamen showed potential for early screening of ANI. | Small sample size with group imbalance; manual segmentation of volume of interest (VOI); limited focus on ANI and pre-clinical ANI, excluding other HAND stages. |
| Salahuddin (2020)^18^ | To improve a prediction model. | Logistic regression. | - χ2 (9)=72.91. - The model explained 35.0% (Nagelkerke R2) of the variance in neurocognitive impairment status and correctly classified 75.8% of cases. | - The identification of predictors, in the Ethiopian people living with HIV, may help in the targeted screening of vulnerable groups during combination antiretroviral therapy (cART) follow-up visits. - This may greatly help in strategizing and implementation of the prevention program, more so, because (i) HIV-associated neurocognitive impairment is an asymptomatic condition for considerable durations, and (ii) clinical trials on neurocognitive impairment therapies have been unsuccessful. | Unable to include HIV- / control participants, not include any psychosocial correlates failed to account for the individual effects of the recreational drugs but reported combined use of khat, coffee, tobacco, and alcohol on neurocognitive functions. |
| Tu (2020)^19^ | To classify neurocognitive status: neurocogn itively normal (NN), HAND, or neurocognitively impaired-other disorders (NCI-OD) and identify key predictive variables for HAND and NCI-OD. | Random Forest (RF) and multiple logistic regression. | - The logistic regression model showed the highest classification accuracy for: - NN vs. HAND=74%; - NN vs. NCI-OD=72%; - HAND vs. NCI-OD=63%. - RF model showed high AUC: - NN vs. HAND=0.87; - NN vs. NCI-OD=0.77; - HAND vs. NCI-OD=0.73; - With high accuracy: - NN vs. HAND=81%; - NN vs. NCI-OD=84%; - HAND vs. NCI-OD=71%. | - HAND prevalence was 21%, with minor neurocognitive disorder (MND) as the most common subtype. - RF model identified key variables, including PHQ-9 score, CD 4 T-cell count, polypharmacy, and sleep hours, for distinguishing neurocognitive groups. | Small sample size for HAND and NCI-OD groups; demographic imbalance (e.g., low proportion of females); overlapping variables between HAND and NCI-OD groups. |
| Underwood (2018)^20^ | To predict baseline and longitudinal cognitive function and to investigate deviations in brain age using MRI data. | Multivariate models using SVM, GPC, KRR, and GPR. | - Baseline prediction: - SVM AUC=0.56; - GPC AUC=0.59. - Baseline regression: - KRR R²=0.08; - GPR R²=0.07. - Longitudinal change prediction was poor: - SVM AUC=0.42; - GPC AUC=0.43. | - Multivariate models were superior to linear models for baseline cognitive function but not clinically useful with poor performance. - Brain-predicted age was slightly older than chronological age in HIV+ individuals and associated with comorbidities and prior AIDS. | Small sample size, heterogeneity in cognitive impairment severity, limited predictive power of MRI data alone for mild impairment, and potential scanner-related variability. |
| Xu (2021)^21^ | To improve diagnostic accuracy of neurocognitive impairment (NCI) classification by combining clinical and imaging features. | SVM with LASSO for feature selection. | - Clinical data only: - AUC=0.62; - Accuracy=0.65; - Sensitivity=0.85; - Specificity=0.37. - MRI data only: - AUC=0.79; - Accuracy=0.72; - Sensitivity=0.86; - Specificity=0.52. - Combined clinical + MRI: - AUC=0.83; - Accuracy=0.80; - Sensitivity=0.86; - Specificity=0.71. | - Combining clinical and MRI features improved classification accuracy and specificity of NCI prediction. - Identified brain regions (e.g., right tapetum, superior frontal gyrus) as predictive of NCI. | Lack of external validation for generalizability. Exclusion of long-term functional outcomes. Not assess daily functional impairment. Potential over-diagnosis with Global Deficit Score (GDS). The possibility of morbidities and coinfections contributing was not accounted for. Since the authors selected MRI features using LASSO, the identified brain regions may not have biological relevance. |
| Yang (2021)^22^ | To identify comorbidity clusters, temporal trend analysis of the prevalence of concurrence of comorbidity clusters. | Hierarchical clustering. | - Not reported. | - This analytic approach helped us identify clinically meaningful groupings from many chronic diseases. - These findings could possibly be generalized to other settings with similar population characteristics (e.g., other Southern US states) and be used to inform the health care systems of the comorbidity clusters occurring within their patient populations. | Comorbidity clustering based on the diagnosis groups may be an artifact of the algorithm. Hierarchical algorithms produce exclusive clusters that one diagnosis group can only exist in one cluster. |
| Zhan (2024)^23^ | To differentiate HIV-ANI from the HIV-normal group. | Logistic regressing. | - Multivariate: - AUC=0.806; - Cutoff Value=0.397; - Sensitivity=72.0%; - Specificity=82.6%. - tCR/Total: - AUC=0.696; - Cutoff Value=0.354; - Sensitivity=56.0%; - Specificity=76.1%. | - Machine Learning methods, specifically logistic regression, demonstrated the potential to classify HIV-ANI based on brain metabolite levels. - The findings emphasize that brain metabolites (e.g., tCr/total) assessed through magnetic resonance spectroscopy (MRS) could be valuable biomarkers for predicting HIV-ANI. - These results suggest the integration of machine learning with neuroimaging data offers promise for improving diagnostic accuracy in neurocognitive disorders. | Small sample size: confounding factors such as recent alcohol consumption and drug use were not considered in the current analysis. |
| Zhang (2022)^24^ | To identify HIV and cognitive impairment statuses using domain-specific learning across multiple cohorts | Multi-label, multi-domain convolutional neural network (CNN) for classification tasks. | - Accuracy=>90% for classification across domains. | - Multi-domain learning improved classification accuracy compared to single-domain approaches. - CNN successfully distinguished HAND from other conditions. | Limited generalizability beyond the datasets included.  Variability in MRI acquisition protocols across cohorts.  Sample size imbalance across labels. |
| Zhang (2016)^25^ | To identify morphometric patterns separating HAND from MCI due to non-HIV conditions in this older age group was proposed. | Logistic regression. | - All patterns except pattern 7 were significant in distinguishing the disease groups, whereas the disease specific classification accuracy for regions was never higher than 68.1%, the lowest accuracy across all patterns was 68.3% for pattern 7 and the highest was 90.8% for pattern 3. | - Simultaneously analyzing all brain regions and time points for disease-specific patterns contributed to distinguishing with high accuracy HAND-related impairment from cognitive impairment found in the HIV uninfected, MCI cohort. | Not explicit mentioned. |
| Zhu (2019)^26^ | To identify symptom clusters. | Principal component analysis, Multiple Linear Model, Association Network. | - Not reported. | - Cognitive dysfunction was the most central symptom cluster. | Only included 27 HIV/AIDS-related symptoms based on three full-scale measurements and one subdomain of a Quality of Life (QOL) scale convenience sampling at five hospital sites, thus, cannot be generalized to the entire Chinese population information about the type of comorbidities was not collected. Also, they only included PLWH who were able to complete the questionnaires by themselves, thus, the level of cognitive status in this sample was relatively high. |

**References**

1. Amusan P, Power C, Gill MJ, et al. Lifetime antiretroviral exposure and neurocognitive impairment in HIV. *Journal of NeuroVirology*. 2020;26(5):743-753. doi:10.1007/s13365-020-00870-z

2. Anderson AE, Jones JD, Thaler NS, Kuhn TP, Singer EJ, Hinkin CH. Intraindividual variability in neuropsychological performance predicts cognitive decline and death in HIV. *Neuropsychology*. 2018;32(8):966-972. doi:10.1037/neu0000482

3. Chockanathan U, Dsouza AM, Abidin AZ, Schifitto G, Wismüller A. Automated diagnosis of HIV-associated neurocognitive disorders using large-scale Granger causality analysis of resting-state functional MRI. *Computers in Biology and Medicine*. 2019;106:24-30. doi:10.1016/j.compbiomed.2019.01.006

4. Am DS, Abidin AZ, Leistritz L, Wismüller A. Identifying HIV Associated Neurocognitive Disorder Using Large-Scale Granger Causality Analysis on Resting-State Functional MRI. *Proc SPIE Int Soc Opt Eng*. 2017;10133doi:10.1117/12.2254690

5. Am DS, Abidin AZ, Wismüller A. Investigating Changes in Resting-State Connectivity from Functional MRI Data in Patients with HIV Associated Neurocognitive Disorder Using MCA and Machine Learning. *Proc SPIE Int Soc Opt Eng*. 2017;10137doi:10.1117/12.2254189

6. Finkelstein A, Faiyaz A, Weber MT, et al. Fixel-Based Analysis and Free Water Corrected DTI Evaluation of HIV-Associated Neurocognitive Disorders. *Frontiers in Neurology*. 2021;12doi:10.3389/fneur.2021.725059

7. Fu D, Mo K, Deng W, et al. Application Value of Machine Learning Method in Measuring Gray Matter Volume of AIDS Patients. *Disease Markers*. 2022;2022doi:10.1155/2022/1210002

8. Gomez D, Power C, Gill MJ, Koenig N, Vega R, Fujiwara E. Empiric neurocognitive performance profile discovery and interpretation in HIV infection. *J Neurovirol*. 2019;25(1):72-84. doi:10.1007/s13365-018-0685-6

9. Levine AJ, Martin E, Sacktor N, Munro C, Becker J. Predictors and Impact of Self-Reported Suboptimal Effort on Estimates of Prevalence of HIV-Associated Neurocognitive Disorders. *Journal of Acquired Immune Deficiency Syndromes*. 2017;75(2):203-210. doi:10.1097/QAI.0000000000001371

10. Luckett P, Wisch J, Cooley SA, Ances BM. Deep-learning cerebral blood flow for cognitive-impairment classification in HIV. *Topics in Antiviral Medicine*. 2019;27(SUPPL 1):46s.

11. Martinez-Banfi M, Vélez JI, Mebarak Chams MR, et al. Utility of a short neuropsychological protocol for detecting HIV-associated neurocognitive disorders in patients with asymptomatic HIV-1 infection. *Brain Sciences*. 2021;11(8)doi:10.3390/brainsci11081037

12. Narvid J, McCoy D, Dupont SM, et al. Abnormal cerebral perfusion profile in older adults with HIV-associated neurocognitive disorder: Discriminative power of arterial spin-labeling. *American Journal of Neuroradiology*. 2018;39(12):2211-2217. doi:10.3174/ajnr.A5902

13. Ogishi M, Yotsuyanagi H. Prediction of HIV-associated neurocognitive disorder (HAND) from three genetic features of envelope gp120 glycoprotein. *Retrovirology*. 2018;15(1)doi:10.1186/s12977-018-0401-x

14. Pinheiro L, Pereira MLD, Fernandez MP, Filho FMV, de Abreu W, Pinheiro P. Application of Data Mining Algorithms for Dementia in People with HIV/AIDS. *Comput Math Methods Med*. 2021;2021:4602465. doi:10.1155/2021/4602465

15. Pulliam L, Liston M, Sun B, Narvid J. Using neuronal extracellular vesicles and machine learning to predict cognitive deficits in HIV. *Journal of NeuroVirology*. 2020;26(6):880-887. doi:10.1007/s13365-020-00877-6

16. Qi Y, Wang W, Rao B, et al. Value of Radiomic Analysis Combined With Diffusion Tensor Imaging in Early Diagnosis of HIV-Associated Neurocognitive Disorders. *Journal of Magnetic Resonance Imaging*. 2023;58(6):1882-1891. doi:10.1002/jmri.28741

17. Qi Y, Xu M, Wang W, et al. Early prediction of putamen imaging features in HIV-associated neurocognitive impairment syndrome. *BMC Neurology*. 2021;21(1):1-10. doi:10.1186/s12883-021-02114-x

18. Salahuddin M, Manzar MD, Hassen HY, et al. Prevalence and Predictors of Neurocognitive Impairment in Ethiopian Population Living with HIV. *HIV AIDS (Auckl)*. 2020;12:559-572. doi:10.2147/hiv.S260831

19. Tu W, Chen PA, Koenig N, et al. Machine learning models reveal neurocognitive impairment type and prevalence are associated with distinct variables in HIV/AIDS. *Journal of NeuroVirology*. 2020;26(1):41-51. doi:10.1007/s13365-019-00791-6

20. Underwood J, Cole JH, Leech R, Sharp DJ, Winston A. Multivariate Pattern Analysis of Volumetric Neuroimaging Data and Its Relationship With Cognitive Function in Treated HIV Disease. *J Acquir Immune Defic Syndr*. 2018;78(4):429-436. doi:10.1097/qai.0000000000001687

21. Xu Y, Lin Y, Bell RP, et al. Machine learning prediction of neurocognitive impairment among people with HIV using clinical and multimodal magnetic resonance imaging data. *J Neurovirol*. 2021;27(1):1-11. doi:10.1007/s13365-020-00930-4

22. Yang X, Zhang J, Chen S, Weissman S, Olatosi B, Li X. Comorbidity patterns among people living with HIV: a hierarchical clustering approach through integrated electronic health records data in South Carolina. *AIDS Care*. 2021;33(5):594-606. doi:10.1080/09540121.2020.1844864

23. Zhan Y, Cai DC, Liu Y, et al. Altered metabolism in right basal ganglia associated with asymptomatic neurocognitive impairment in HIV-infected individuals. *Heliyon*. 2024;10(1):e23342. doi:10.1016/j.heliyon.2023.e23342

24. Zhang J, Zhao Q, Adeli E, et al. Multi-label, multi-domain learning identifies compounding effects of HIV and cognitive impairment. *Medical Image Analysis*. 2022;75doi:10.1016/j.media.2021.102246

25. Zhang Y, Kwon D, Esmaeili-Firidouni P, et al. Extracting patterns of morphometry distinguishing HIV associated neurodegeneration from mild cognitive impairment via group cardinality constrained classification. *Human Brain Mapping*. 2016;37(12):4523-4538. doi:10.1002/hbm.23326

26. Zhu Z, Hu Y, Xing W, et al. Identifying Symptom Clusters Among People Living With HIV on Antiretroviral Therapy in China: A Network Analysis. *Journal of Pain and Symptom Management*. 2019;57(3):617-626. doi:10.1016/j.jpainsymman.2018.11.011
